# Pediatric Subligamentous Cordectomy: Successful Voice Outcome Following Laryngeal Granular Cell Tumor Resection

**DOI:** 10.1155/crot/5734361

**Published:** 2025-08-01

**Authors:** Jillian L. Haywood, Katherine Guo, Nicholas Toomey, Tiffiny A. Hron, Alexander P. Marston

**Affiliations:** ^1^Department of Anesthesiology, Tufts University School of Medicine, Boston, Massachusetts, USA; ^2^Department of Otolaryngology-Head and Neck Surgery, UC Davis Health, Sacramento, California, USA; ^3^Center for Laryngeal Surgery & Voice Rehabilitation, Harvard Medical School, Massachusetts General Hospital, Boston, Massachusetts, USA

**Keywords:** granular cell tumor, pediatric vocal cord lesion, subligamentous cordectomy

## Abstract

**Background:** Laryngeal granular cell tumors (GCTs) are rare lesions, particularly in pediatric patients. We present a case of GCT of the right true vocal cord in a 12-year-old male.

**Methods:** The electronic medical record was utilized to review the patient's clinical and surgical history.

**Results:** A 12-year-old male presented with a 2-year history of worsening dysphonia and cough. Flexible laryngoscopy revealed a submucosal pedunculated mass of the right mid and anterior true vocal cord. Gross resection was performed. Final pathology confirmed a benign GCT; however, a positive margin was identified. A right subligamentous cordectomy was pursued for complete excision of the residual GCT. The patient achieved an excellent voice outcome.

**Conclusions:** This case demonstrates that subligamentous cordectomy can be an effective treatment method for laryngeal GCT in pediatric patients. It also adds valuable data regarding pediatric GCT location and propensity for submucosal tumor spread.

## 1. Introduction

Granular cell tumors (GCTs) are rare, usually benign tumors of Schwann cell origin that arise in various tissue types. Laryngeal GCTs in pediatric patients are rare with a recent literature review identifying only 38 documented cases [1]. Malignant transformation and airway obstruction are uncommon but potentially life threatening [2]. Progressive dysphonia is common with a secondary reduction in quality of life. Due to limited data, characteristic features of pediatric GCTs have not been entirely defined [3]. As a result, standardized treatment and surveillance algorithms do not exist. Given their infiltrative nature, an effort to delineate the most effective treatments to prevent recurrence is necessary. This report presents a case of a GCT of the right vocal cord in a 12-year-old male treated initially with gross resection of the lesion, followed by a subligamentous cordectomy for excision of residual tumor.

## 2. Case Presentation

**Figure 1:**
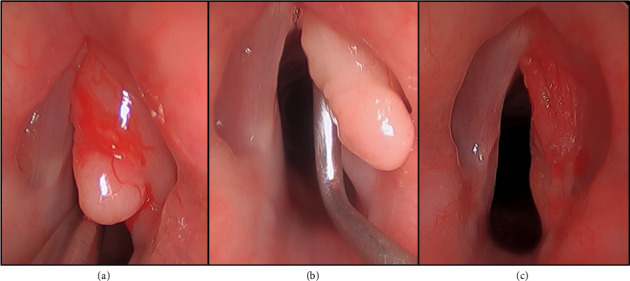
Direct laryngoscopy image demonstrating submucosal pedunculated mass of the right mid and anterior vocal cord (a-b). Immediate postoperative result status: post microflap elevation with gross mass excision using the CO2 laser (c).

**Figure 2:**
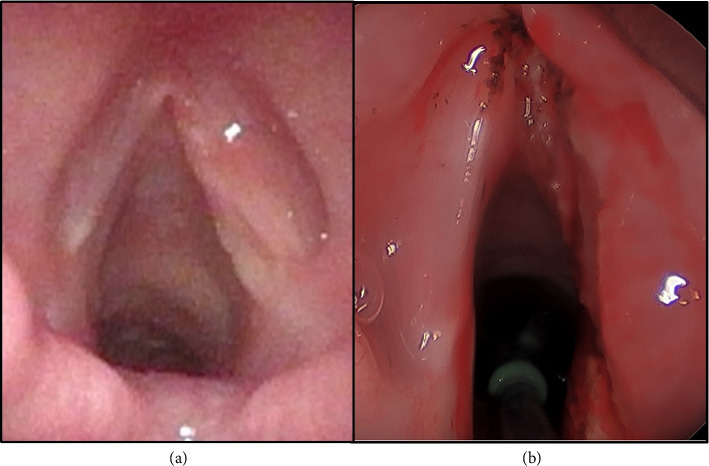
Flexible laryngoscopy image demonstrating persistent granular cell tumor along the length of the right true vocal fold (a). Immediate postoperative direct laryngoscopy image status: post right subligamental cordectomy for resection of residual granular cell tumor (b).

A 12-year-old previously healthy male presented with a 2-year history of worsening dysphonia and cough without dyspnea. Flexible laryngoscopy revealed a large right-sided white submucosal vocal cord mass without subglottic or supraglottic involvement. Videostroboscopy demonstrated an absent mucosal wave with phonation on the involved side. The patient subsequently underwent a direct laryngoscopy which demonstrated a pedunculated submucosal mass of the right mid and anterior vocal cord (Figures 1(a) and 1(b)). A microflap was elevated and the mass was grossly excised with a CO2 laser (Figure 1(c)). Intraoperative frozen section pathologic analysis was consistent with a benign lesion. Ultimately, the final pathology confirmed a benign GCT with a positive posterior vocal fold margin. The patient experienced improved but persistent dysphonia over the next several months. Follow-up videostroboscopy showed persistent GCT with interval growth along the right posterior vocal fold (Figure 2(a)) without mucosal vibration during phonation.

Ten months after the initial excision of the tumor, the patient underwent suspension laryngoscopy with KTP-assisted right subligamentous cordectomy for GCT excision. Intraoperatively, the tumor was found to encompass the entire length of the right vocal fold from the anterior commissure to the vocal process. There was no involvement of the paraglottic space and the tumor did not involve the cricoarytenoid joint. The depth of the tumor was to the level of the vocal ligament with a significant infracordal component. The resection was carried through the vocal ligament down to the vocalis muscle for both an oncologic margin and to potentially facilitate thyroarytenoid muscle vibration during phonation (Figure 2(b)). Frozen section pathologic analysis was completed and negative margins were obtained.

**Figure 3:**
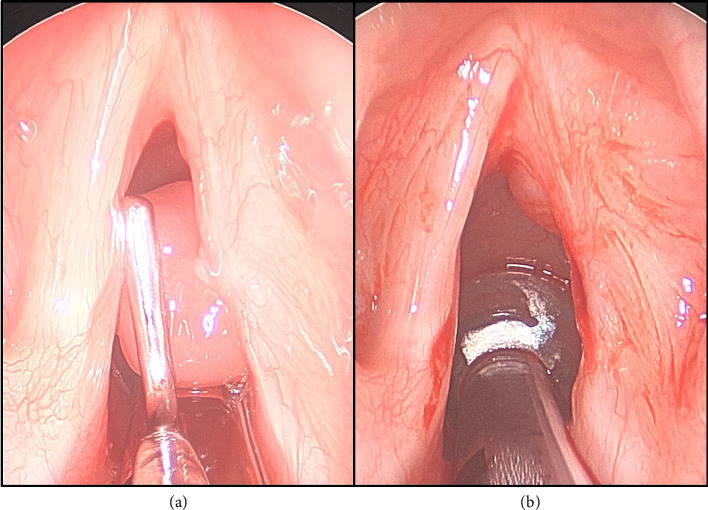
Direct laryngoscopy view showing a pedunculated vocal fold polyp (a). Immediate post polyp excision using the KTP laser (b).

**Figure 4:**
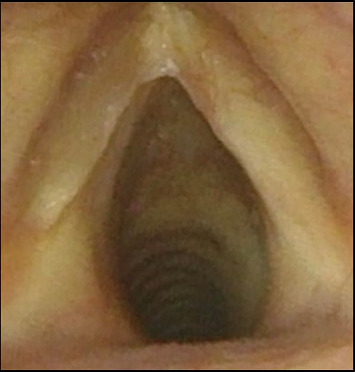
Flexible laryngoscopy view 16 months' status: post polyp excision revealing a well-healed right true vocal fold without evidence of recurrent tumor.

Three months postoperatively following the right subligamentous cordectomy, flexible fiberoptic laryngoscopy revealed an 8 mm pedunculated polypoid granuloma emanating from the medial surface of the midaspect of the right true vocal fold. The lesion persisted during ongoing surveillance visits and was ultimately excised via direct laryngoscopy with pathologic confirmation of a granuloma (Figures 3(a) and 3(b)). Postexcision, the right vocal fold re-epithelialized without recurrence of the granuloma. Videostroboscopy evaluation 2 years later revealed the presence of a mucosal wave with phonation and there was no evidence of recurrent GCT (Figure 4). Furthermore, the patient described a significantly improved voice and cough resolution. The GRBAS scale was used as a perceptual assessment of voice quality [4]. With this scale, the grade, roughness, breathiness, asthenia, and strain of the patient's voice was assessed by the senior author prior to the initial surgery and at the last 2-year follow-up. Preoperatively, the GRBAS scale composite score was 7 (Grade = 2, Roughness = 2, Breathiness = 1, Asthenia = 1, and Strain = 1). Postoperatively, the GRBAS scale composite score was 5 (Grade = 1, Roughness = 1, Breathiness = 1, Asthenia = 1, and Strain = 1).

## 3. Discussion

This patient initially presented with dysphonia and cough of multiple years duration despite seeking primary medical care around the time of symptom onset. The prolonged time-to-diagnosis highlights the insidious nature of laryngeal GCT and underscores the importance of visualization and biopsy of the mass. Dysphonia, as was the chief complaint of the presented patient, is the most frequently reported presenting symptom of pediatric laryngeal GCT, affecting 81% of the patients [1].

Unlike some laryngeal masses, GCTs are neither sensitive to radiotherapy nor chemotherapy. Therefore, surgical management is considered the gold standard and complete resection with negative margins is recommended due to a low risk of malignancy and a high risk of recurrence. In a study by Ahmed et al. the extent of surgical resection is a critical predictor of prognosis in GCTs of the sellar region. Gross total resection (GTR) is associated with better overall survival compared with subtotal resection (STR) or biopsy alone, supporting the recommendation for complete resection with negative margins [5]. However, because of limited data, the specifics of surgical management are not well defined. In the 38 pediatric laryngeal GCT cases identified by Mur et al., 61% were treated endoscopically, 33% underwent a laryngofissure for tumor excision, and one patient each underwent partial laryngectomy and total laryngectomy. It is estimated in adult patients that GCT recurrence is approximately 8% if negative margins are obtained. That risk increases to 21%–50% when the margins are positive [1]. However, due to the benign nature of the lesion and low potential for malignant transformation, patient-specific factors and the severity of GCT disease burden are important to consider when devising the optimal surgical plan [6].

The goal for both surgeries in the 12-year-old male presented in this case was for complete tumor excision to optimize the voice outcome and mitigate the risk of malignancy. Roh et al. showed that in adults with glottic carcinoma, subepithelial or subligamentous cordectomies have better voice outcomes than transmuscular, total, or extended cordectomies [7]. Furthermore, Hillel et al. showed that those who underwent subligamentous excision reported superior voice outcomes than those who underwent subepithelial cordectomies [8]. These patients also had improved mucosal wave scores. The authors concluded that a subligamentous cordectomy is advised if the tumor resection reaches the level of the vocal ligament. Regenerated epithelium over ligament does not vibrate. However, regenerated epithelium over muscle has the potential to vibrate. Since in the presented case, the GCT involved the entire length of the vocal fold to a depth of the vocal ligament, the decision to remove the ligament was made in hopes of achieving an improved voice outcome. Two years postoperatively, the patient had no evidence of tumor recurrence with an excellent voice outcome measured by the GRBAS scale and evidence of a mucosal wave during phonation on videostroboscopy.

## 4. Conclusion

This case demonstrates that a subligamentous cordectomy technique can be an effective treatment method for pediatric laryngeal GCTs in cases of recurrence and/or with tumor involvement to the level of or involving the vocal ligament. It can potentially allow for attaining a safe surgical margin with the potential for an improved postoperative voice outcome. In the presented case, this method of laryngeal GCT resection facilitated complete tumor resection without evidence of recurrence and a strong voice outcome.

## Data Availability

Data sharing is not applicable to this article as no new data were created or analyzed in this study.
